# Transmembrane transport of fluoride studied by time-resolved emission spectroscopy†

**DOI:** 10.1039/d3cc00897e

**Published:** 2023-03-14

**Authors:** Alessio Cataldo, Matúš Chvojka, Gyeongjin Park, Vladimír Šindelář, François P. Gabbaï, Stephen J. Butler, Hennie Valkenier

**Affiliations:** a Université libre de Bruxelles (ULB), Engineering of Molecular NanoSystems, Avenue F.D. Roosevelt 50, CP165/64 B-1050 Brussels Belgium hennie.valkenier@ulb.be; b Department of Chemistry and RECETOX, Faculty of Science, Masaryk University Brno 62500 Czech Republic; c Department of Chemistry, Texas A&M University College Station Texas 77843 USA; d Department of Chemistry, Loughborough University, Epinal Way Loughborough UK s.j.butler@lboro.ac.uk

## Abstract

Here we present a new method to monitor fluoride transmembrane transport into liposomes using a europium(iii) complex. We take advantage of the long emission lifetime of this probe to measure the transport activity of a fluorescent transporter. The high sensitivity, selectivity, and versatility of the assay allowed us to study different types of fluoride transporters and unravel their mechanisms of action.

## Abstract 2

The encapsulation of a lanthanide-based probe in liposomes allows monitoring of fluoride transmembrane transport using time-resolved emission spectroscopy, which makes it possible study fluorescent transporters.

## Graphical abstract

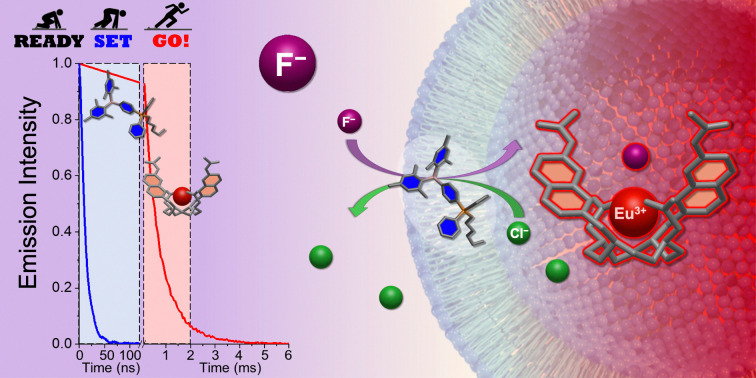

The frequent use of fluoride as an anti-caries agent in oral hygiene products and in drinking water has resulted in significant health benefits.^1^ On the other hand, fluoride is potentially toxic at mM concentrations due to its ability to penetrate cells and inhibit phosphoryl-transfer enzymes required for energy production and nucleic acid synthesis.^1,2^ Many bacteria are equipped with natural fluoride channels that can export the anion out of the cells allowing survival in the presence of high doses of fluoride.^3^ These findings have been the driving force for the development of synthetic transmembrane transporters^4^ capable of transporting fluoride through cell membranes.^5–10^ Such transporters could, for instance, have antibacterial properties.^11^ After the first encouraging results from the Matile group,^5^ the Gale group reported a series of strapped calix[4]pyrroles that function as synthetic fluoride carriers.^6^ Recently, Gabbaï and co-workers reported molecules 1^8^ and 2^7^ (Fig. 1), that can bind fluoride *via* Lewis acid–base interactions and shuttle this anion through the lipidic membrane of liposomes. These reported fluoride transporters were studied using either indirect methods^5,10^ or an ion selective electrode (ISE) to study fluoride uniport.^6–9^

**Fig. 1:**
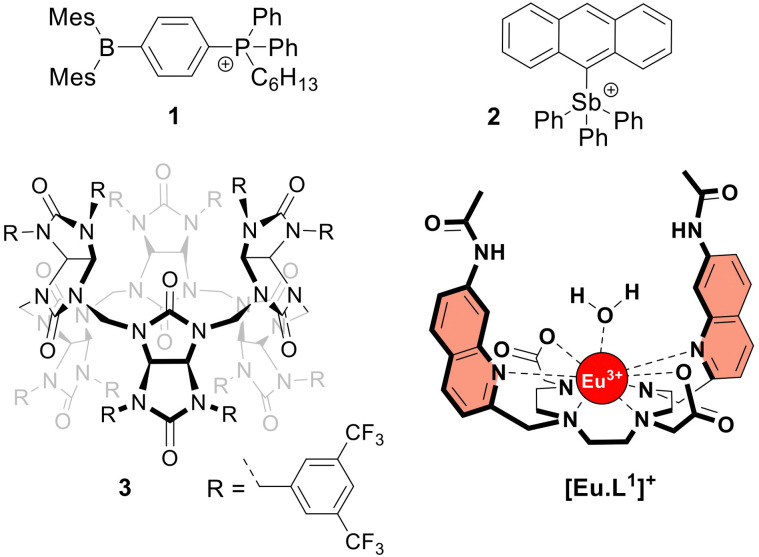
Structures of the investigated fluoride transporters (1–3) and the emissive europium probe [Eu·L^1^]^+^.

However, these methods have limitations in sensitivity, selectivity, and versatility that have impeded more extensive studies on the transmembrane transport of fluoride. To overcome these limitations, monitoring the emission of a fluoride-sensitive probe encapsulated in liposomes is particularly promising because of the high sensitivity of this approach. For this purpose, luminescent lanthanide probes offer unique photophysical advantages over classical organic fluorescent probes including long emission lifetimes that enable time-resolved measurements, thereby increasing precision and signal to noise ratio.^12^ The hydrophilic europium(iii) complex [Eu·L^1^]^+^ (Fig. 1), previously developed by Butler^13^ and recently employed to study the transmembrane transport of bicarbonate,^14^ was identified as a good candidate for monitoring fluoride transport. Upon exciting the organic ligand at 332 nm, the complex [Eu·L^1^]^+^ emits light from the Eu(iii) ion in the 570–720 nm wavelength range. The binding of fluoride to the Eu(iii) ion displaces the coordinated water causing a significant enhancement in emission intensity and a change in the emission spectral fingerprint of the probe. The emission lifetime of the [Eu·L^1^]^+^ probe, which increases from 0.49 ms to 1.13 ms upon binding fluoride in water,^13^ allows time-resolved measurements to be performed^15–17^ to eliminate short-lived fluorescence arising from organic fluorophores, such as the biomolecules present in cells or synthetic ion transporters.^18,19^

Here we present a time-resolved luminescence assay to directly monitor the influx of fluoride into liposomes. We demonstrate that the transport of anions by fluorescent anion transporters can be monitored without interference from the transporter itself. Moreover, the combined use of the time-resolved EuL1 assay with the existing HPTS assay enabled the different mechanisms of fluoride transport by compounds 1–3 to be distinguished.

To monitor the transmembrane transport of fluoride, the complex [Eu·L^1^]^+^ was first encapsulated into large unilamellar vesicles (LUVs, ∼200 nm diameter) suspended in a 225 mM NaCl solution (to promote F^−^/Cl^−^ exchange) buffered at pH 7 using 0.5 mM HEPES (Fig. 2a). Transporters 1 and 2 were post-inserted into the liposomal membrane using MeOH solutions before starting the experiment, while transporter 3 was pre-incorporated in the membrane during the preparation of the liposomes, due to its high lipophilicity resulting in poor deliverability.^20^ A 3 mM pulse of NaF was added to the LUVs after 30 seconds and the liposomes were lysed by addition of Triton X-100, 600 seconds after the addition of NaF. Detailed procedures and the optimisation of the assay can be found in the ESI.†

**Fig. 2:**
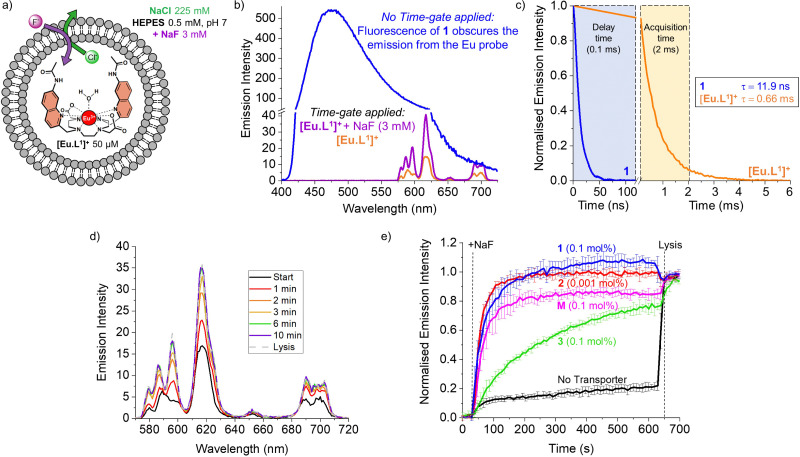
(a) Schematic representation of the EuL1 assay to monitor the transport of fluoride; (b) emission spectra of LUVs with [Eu·L^1^]^+^ encapsulated (50 μM) and transporter 1 incorporated in the lipid bilayer (0.1 mol%) when excited at 332 nm without time-gating (blue spectrum) and with time-gating in the absence (orange) and in presence of 3 mM NaF (purple). (c) Time gating parameters selected based on the short emission lifetime of transporter 1 (0.1 mol%) incorporated in the lipid bilayer of LUVs (blue curve) *versus* the long emission lifetime of complex [Eu·L^1^]^+^ encapsulated in LUVs (orange curve); (d) emission spectra of [Eu·L^1^]^+^ recorded during the transport by 1 (at 0.1 mol%); (e) normalised transport curves of transporters 1–3 and monensin (M), obtained in the EuL1 assay monitoring the emission intensity at 615 nm upon excitation at 332 nm, with a time-gate applied (delay time 0.1 ms, acquisition time 2 ms).

During the experiment, the liposome suspension was irradiated at 332 nm and the europium emission band at 615 nm was monitored, as this showed the largest emission increase upon addition of fluoride. When phosphonium borane 1 was added to the lipid bilayer of LUVs containing the complex [Eu·L^1^]^+^, excitation at 332 nm gave rise to a large, broad emission band ranging between 400 and 700 nm, produced by the emissive properties of compound 1 (Fig. 2b, blue spectrum). The sharp emission bands of the europium complex were completely obscured by the broad fluorescence of 1, preventing the study of transmembrane transport of fluoride mediated by compound 1 using steady-state fluorescence spectroscopy.

A potential solution to this problem is the use of time-resolved measurements.^15,21^ This method takes advantage of the long emission lifetime of [Eu·L^1^]^+^ and the use of a pulsed light source in combination with control over the recording delays by the detector. After the irradiation pulse, the detector does not record until the unwanted background fluorescence (*e.g.* of transporter 1) has ceased. After this delay, the detector records the emission only from the europium probe. This strategy opens the possibility of studying transporters that exhibit fluorescence that interferes with the transport measurements. To assess the viability of such time-resolved measurements, the lifetimes of [Eu·L^1^]^+^ and compound 1 were determined (Fig. 2c, see ESI† for details).

The probe [Eu·L^1^]^+^ showed a lifetime of 0.66 ms when encapsulated in LUVs, significantly longer than the emission lifetime of compound 1, which was found to be 11.9 ns. Thus, using a delay of 0.1 ms followed by 2 ms acquisition time, we were able to eliminate interference from compound 1 and selectively observe the emission bands of [Eu·L^1^]^+^ before and after the addition of NaF (Fig. 2b, orange and purple spectra). To ensure consistency, time-resolved measurements were used also in experiments with non-fluorescent transporters.^22^

The increase in emission intensity of [Eu·L^1^]^+^ upon addition of NaF in the absence of a transporter is minimal (Fig. 2e, black curve), confirming the efficient encapsulation of the probe. For transporters 1, 2 and 3, the addition of a 3 mM NaF pulse results in a clear increase of the emission (Fig. 2d and e), which suggests that these transporters can promote fluoride transport across the liposomal membrane and highlights the sensitivity of our new method.^23^ Transporter 2 has the highest activity, as apparent from the rapid increase of the curve (0.001 mol%, initial rate *I* = 0.10 s^−1^), even though a 100-fold lower concentration of 2 was used compared to the other transporters. Transporter 1 (0.1 mol%, *I* = 0.063 s^−1^) still gives rise to full equilibration of the fluoride gradient in less than 600 seconds, at a 20-fold lower concentration compared to that needed in the ISE assay.^8^ Furthermore, despite the reported poor activity of 1 as a Cl^−^ uniporter,^8^ our time-resolved EuL1 assay reveals that 1 is a good F^−^/Cl^−^ antiporter. Transporters 1 and 2 were studied at a range of different concentrations, which allowed us to determine their EC_50,600s_ values (*i.e.*, the concentration at which half of the maximal transport response after 600 s is obtained) to be 0.009 mol% for 1 and 0.000014 mol% for 2 (Fig. S6 and S7, ESI†). Compound 3 is a fluorinated bambusuril, which was previously reported as an efficient HCO_3_^−^/Cl^−^ antiporter^14,20^ and could not be tested as fluoride transporter by previously reported methods due to its poor deliverability. We preincorporated compound 3 in the LUVs, tested it as F^−^/Cl^−^ transporter, and found mild activity (0.1 mol%, *I* = 0.011 s^−1^).

While the experimental conditions in this EuL1 assay would favour F^−^/Cl^−^ antiport (Fig. 3a), different mechanisms can give rise to a transport response. Although F^−^ cannot diffuse spontaneously across a lipidic membrane, the neutral molecule HF can easily diffuse until a pH gradient is built up (Fig. S3, ESI†). The presence of a transporter able to equilibrate a pH gradient would prevent the acidification of the interior and promote diffusion of HF into the liposomes, causing an emission increase leading to apparent fluoride transport (Fig. 3b and c). Thus, when the potent cationophore monensin^6^ was tested as transporter in the EuL1 assay, a clear increase of emission was observed due to Na^+^/H^+^ antiport and HF diffusion into the liposomes (Fig. 2e, magenta curve). This means that a transporter able to dissipate a pH gradient across the lipidic membrane by OH^−^/Cl^−^ antiport (or equivalent H^+^/Cl^−^ symport^24^) could also show activity in the EuL1 assay even without being directly involved in the transport of fluoride anions (Fig. 3c).

**Fig. 3:**
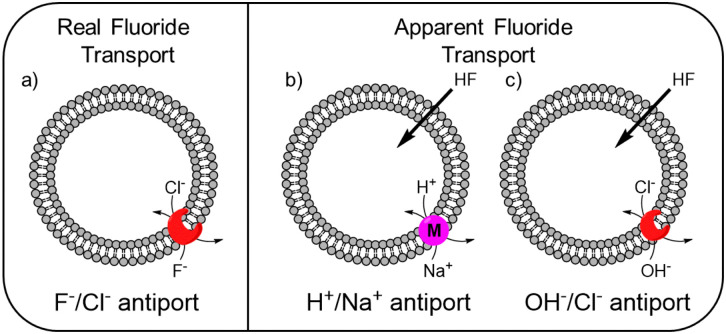
Schematic representation of the mechanisms linked to real (a) and apparent (b and c) transport of fluoride.

For this reason, we used the pH-sensitive probe HPTS^25^ and a pH gradient to monitor the OH^−^/Cl^−^ antiport activity of transporters 1–3 (see ESI† for experimental details).^24^ Transporter 2 was found to be highly active in OH^−^/Cl^−^ antiport at 0.001 mol%, among the best OH^−^/Cl^−^ or equivalent H^+^Cl^−^ transporters reported to date.^11,26^ On the other hand, transporter 1 showed low activity and 3 showed no activity as OH^−^/Cl^−^ antiporter, even when tested at 0.1 mol% (Fig. S16 and S17, ESI†).

As the HPTS and EuL1 assays are performed under similar conditions, the direct comparison of the transport curves acquired in the two assays gives a clear indication of the F^−^/Cl^−^ antiport ability of the transporters. The comparison of the transport curves obtained in the two assays for 1 (Fig. 4a) reveals a much faster response in the EuL1 assay, which implies a strong predominance of F^−^/Cl^−^ antiport over OH^−^/Cl^−^ antiport, while the latter combined with HF could still contribute to part of the emission response in the EuL1 assay. In contrast, the transport curves obtained for compound 2 in the two assays are more similar (Fig. 4b), suggesting that OH^−^/Cl^−^ antiport combined with HF diffusion is the main mechanism (∼70%) and that true fluoride transport contributes to a lower extent (∼30%). Notably, transporter 3 did not show any activity in the HPTS assay but a clear response in the EuL1 assay, thus confirming that it works exclusively as a F^−^/Cl^−^ antiporter (Fig. 4c).

**Fig. 4:**
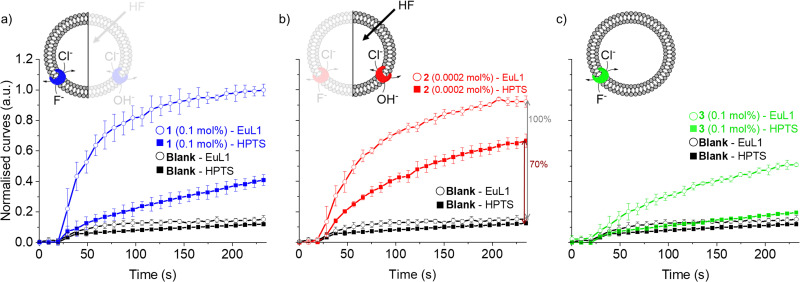
Direct comparison of transport curves obtained in the HPTS (filled symbol) and EuL1 (open symbols) assays for transporters 1 (a), 2 (b) and 3 (c).

Our new assay is not limited to the study of F^−^/Cl^−^ antiport; changing the salt in the buffer gives the possibility to study F^−^/NO_3_^−^ antiport and F^−^ uniport. Furthermore, the time-resolved assay presented here can also be used to study the transport of other anions than F^−^, such as HCO_3_^−^ (Fig. S20, ESI†). All three transporters were active as F^−^/NO_3_^−^ antiporters (Fig. S8, ESI†), although the involvement of OH^−^/NO_3_^−^ antiport mechanism cannot be excluded. While 2 was not active as F^−^ uniporter (at 0.001 mol%, the concentration at which it is highly active as antiporter), 1 showed mild activity and 3 was the most active (both at 0.1 mol%) when combined with cationophore valinomycin in a potassium gluconate solution (Fig. S10, ESI†). The activity of the three transporters to work as Cl^−^ uniporters and OH^−^ uniporters was also tested and revealed efficient Cl^−^ uniport by 3, while none of the transporters showed significant OH^−^ uniport activity at the above-mentioned concentrations (Fig. S16 and S19, ESI†). Compound 2 was previously reported to be active as uniporter for different anions (OH^−^, Cl^−^, and F^−^) at 1–2 mol%,^7^ while we found it to be a highly active antiporter (F^−^/Cl^−^ and OH^−^/Cl^−^) at 0.001 mol%, indicating that the overall neutral complex of 2 with an anion crosses the membrane much more readily than the positively charged transporter 2 itself.

In conclusion, we have developed a time-resolved emission spectroscopy assay to directly monitor the transmembrane transport of fluoride by synthetic transporters. The method overcomes the limitations of the ion selective electrode assay and other indirect methods (see Table S1, ESI†), providing high selectivity for F^−^ anions and allowing the study of F^−^/Cl^−^ and F^−^/NO_3_^−^ antiport processes along with F^−^ uniport. Time-resolved measurements were used for the first time to study anion transmembrane transport mediated by fluorescent compounds, highlighting the advantage of using lanthanide probes to study transmembrane transport processes.

We thank Dr Ludovic Troian-Gautier for the measurement of the emission lifetime of transporter 1. AC is a FRIA grantee, MC a Research Fellow, and HV a Research Associate of the Fonds de la Recherche Scientifique – FNRS. HV acknowledges the European Research Council (ERC, Grant agreement 802727) for funding, SJB the Engineering and Physical Sciences Research Council (EPSRC, EP/S032339/1), FPG the National Science Foundation (CHE-2108728) and the Welch Foundation (A-1423), and V. Š. the Czech Science Foundation (No. GA20-13922S).

## Conflicts of interest

There are no conflicts to declare.

## Supplementary Material

CC-059-D3CC00897E-s001
