# Supplementary material for: Transmembrane transport of fluoride studied by time-resolved emission spectroscopy
Source: Chem Commun (Camb). 2023 Mar 14;59(28):4185–8. doi: 10.1039/d3cc00897e (PMC10072081; doi:10.1039/d3cc00897e)
Supplement: CC-059-D3CC00897E-s001 [file CC-059-D3CC00897E-s001.pdf]

Electronic Supplementary Material (ESI) for ChemComm.
This journal is © The Royal Society of Chemistry 2023

Electronic Supplementary Information

Transmembrane Transport of Fluoride Studied by Time-Resolved Emission Spectroscopy

Alessio Cataldo,[a] Matúš Chvojka,[a,b] Gyeongjin Park,[c] Vladimír Šindelář,[b] François P. Gabbaï,[c] Stephen J. Butler,*[d] Hennie Valkenier*[a]

[a] *Université libre de Bruxelles (ULB), Engineering of Molecular NanoSystems, Avenue F.D. Roosevelt 50, CP165/64, B-1050 Brussels.*
[b] *Department of Chemistry and RECETOX, Faculty of Science, Masaryk University, Brno 62500, Czech Republic.*
[c] *Department of Chemistry, Texas A&M University, College Station, Texas 77843, USA.*
[d] *Department of Chemistry, Loughborough University, Epinal Way, Loughborough, UK.*

E-mail: hennie.valkenier@ulb.be
E-mail: s.j.butler@lboro.ac.uk

Table of Contents

1. Comparison of the EuL1 assay and the ISE assay for the study of transmembrane transport of fluoride ... 3

2. General procedure for the preparation of liposomes ... 4

2.1 DLS Analysis of the Large unilamellar Vesicles ... 6

3. EuL1 assay ... 7

3.1 General procedure for transport measurements with EuL1 assay ... 7

3.2 Optimization of the EuL1 assay ... 8

3.3 Quantification of rates of transport and half-lives. ... 11

3.4 EC_{50} calculation in the EuL1 assay to monitor F^{-}/Cl^{-} antiport ... 12

3.5 EuL1 assay to monitor F^{-}/NO_3^{-} antiport ... 13

3.6 Study of transmembrane transport of fluoride in potassium chloride ... 14

3.7 EuL1 assay to monitor F^{-} uniport ... 15

4. Lifetime measurements and application of time-resolved measurements ... 16

4.1 Lifetime of $[Eu.L^1]^+$ encapsulated in LUVs ... 16

4.2 Lifetime of compound **1** incorporated in liposomes ... 17

4.3 EuL1 assay to study fluoride transport without time-gating ... 18

5. HPTS assay ... 19

5.1 HPTS experiment with NaF pulse to prove pH build-up ... 19

5.2 General procedure for transport measurements with HPTS ... 21

5.3 HPTS assay to monitor OH^{-}/Cl^{-} antiport and Cl^{-} uniport ... 22

5.4 EC_{50} calculation in the HPTS assay to monitor the OH^{-}/Cl^{-} antiport ... 23

5.5 Comparison of the EuL1 and HPTS assays ... 24

5.6 HPTS assay to monitor OH^{-} uniport ... 25

6. Application of the time-resolved EuL1 assay for the study of bicarbonate transport ... 26

References ... 27

1. Comparison of the EuL1 assay and the ISE assay for the study of transmembrane transport of fluoride

Table S1 Comparison of the assay presented in this work with the commonly used ISE assay[1-4]

Criteria	Definition	Fluoride Selective Electrode	The EuL1 assay presented here
Sensitivity	Fluoride gradient used	300 mM	3 mM
Versatility *and* ***Selectivity***	Transport mechanisms studied	F^- uniport	F^-/Cl^- antiport<br>F^-/NO_3^- antiport<br>F^- uniport
Versatility	Study of highly lipophilic transporters, such as compound **3**	No (only post-insertion)	Yes (post-insertion and pre-incorporation)
Mechanism	Possibility to distinguish *apparent* and *real* transport of fluoride and to compare *uniport* and *antiport* mechanisms	No	Yes

2. General procedure for the preparation of liposomes

Stock solutions of 2-oleoyl-1-palmitoyl-*sn*-glycero-3-phosphocholine (POPC), (purity > 99%) and cholesterol, (purity > 95%) were prepared in freshly deacidified chloroform and used for the preparation of the liposomes. Transporters **1**-**3** and probe $[Eu.L^1]^+$ have been synthesised according to previously published procedures.[3,5-7] Buffer solutions were prepared in water that was deionized with a Millipore filtration system.

A solution with a 7:3 POPC:cholesterol ratio was prepared using volumes calculated to obtain a final lipids concentration of 0.4 mM (for EuL1 assay) or 0.1 mM (for HPTS assay). When **3** was employed as transporter, a 0.1 mM solution in acetonitrile was combined to the lipids solution to reach a concentration of 0.1 mol% (transporter to lipids molar percentage). The solvent was evaporated under a flow of nitrogen and the resulting lipid film was dried under vacuum for at least 1 hour.

Depending on the method used, the lipid films were hydrated with:

a) <u>EuL1 assay for the study of F^-/Cl^- antiport in NaCl</u>
500 µL of an aqueous solution of the probe $[Eu.L^1]^+$ (50 µM, prepared from a 5 mM stock solution in methanol) in NaCl (225 mM) and HEPES (0.5 mM) buffered at pH 7.

b) <u>EuL1 assay for the study of F^-/Cl^- antiport in KCl</u>
500 µL of an aqueous solution of the probe $[Eu.L^1]^+$ (50 µM, prepared from a 5 mM stock solution in methanol) in KCl (225 mM) and HEPES (0.5 mM) buffered at pH 7.

c) <u>EuL1 assay for the study of F^-/NO_3^- antiport</u>
500 µL of an aqueous solution of the probe $[Eu.L^1]^+$ (50 µM, prepared from a 5 mM stock solution in methanol) in $NaNO_3$ (225 mM) and HEPES (0.5 mM) buffered at pH 7.

d) <u>EuL1 assay for the study of F^- uniport</u>
500 µL of an aqueous solution of the probe $[Eu.L^1]^+$ (50 µM, prepared from a 5 mM stock solution in methanol) in potassium gluconate (KGluc, 100 mM) and HEPES (0.5 mM) buffered at pH 7.

e) <u>HPTS assay (for the study of OH^-/Cl^- antiport and Cl^- uniport)</u>
500 µL of an aqueous solution of HPTS (1 mM) in $NMDGH^+Cl^-$ (100 mM) and HEPES (10 mM) buffered at pH 6.8. The buffer solution was prepared by mixing a 1.0 M solution of *N*-methyl-ᴅ-glucamine (NMDG) with the appropriate amounts of HCl, HEPES, and water to reach the desired concentrations and pH.

f) <u>HPTS assay (for the study of OH^- uniport)</u>
500 µL of an aqueous solution of HPTS (1 mM) in sodium gluconate (NaGluc, 100 mM) and HEPES (10 mM) buffered at pH 7.

The hydrated lipid films were sonicated for ca. 30 s and stirred for 1 hour at room temperature to create a heterogeneous mixture of vesicles. The suspension was subjected to 10 freeze-thawing cycles to generate unilamellar vesicles, diluted to 1 mL with the same salt solution previously employed for the hydration of the lipid film (without the fluorescent probe) and extruded 29 times through a polycarbonate membrane (200 nm pore size) at room temperature. The large unilamellar vesicles (LUVs) suspension was eluted through a size exclusion column (Sephadex G-25) using the external salt solution as eluent to remove the external fluorescent probe. The resulting suspension was further diluted with the same salt solution to obtain the desired final lipids concentration of 0.4 mM (for the EuL1 assay) or 0.1 mM (for the HPTS assay) assuming no loss of lipids.

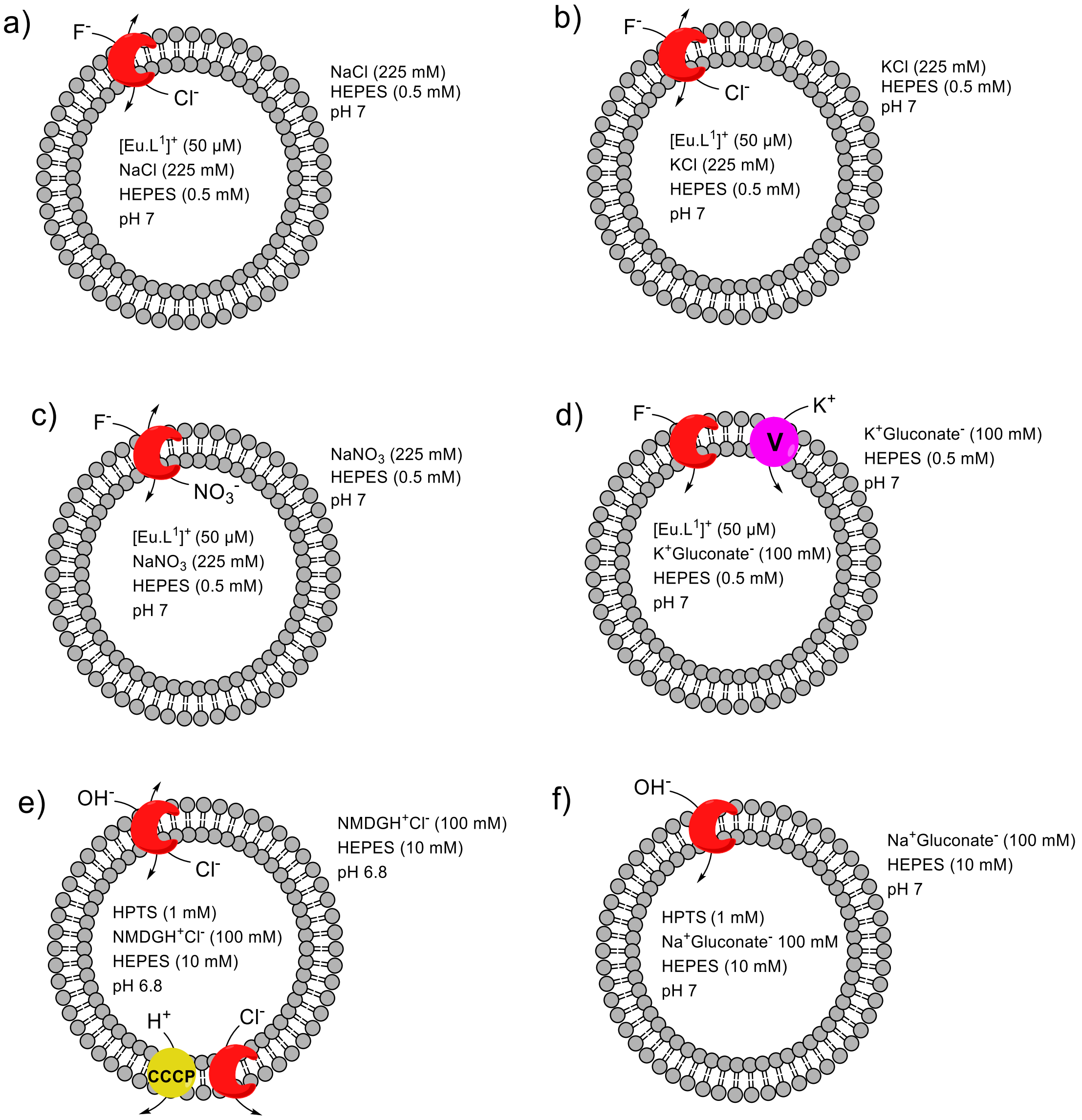

Figure S1. Schematic representation of the conditions used to monitor the transport of fluoride using the EuL1 assay (a-d) and the transport of chloride (e) and hydroxide (f) using the HPTS assay.

2.1 DLS Analysis of the Large unilamellar Vesicles

DLS data from 4 different batches of LUVs (prepared as described in Section 2a) are plotted below. An average of 180 nm was found for the hydrodynamic diameter of the LUVs, as is common for liposomes after extrusion through a polycarbonate membrane with 200 nm pores.

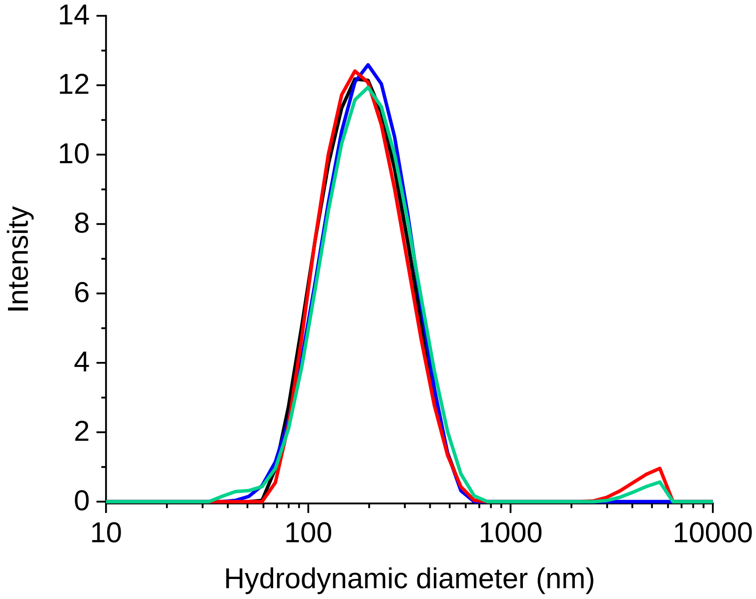

Figure S2. DLS data for 4 batches of LUVs with probe $[Eu.L^1]^+$ encapsulated and suspended in 225 mM NaCl with 0.5 mM HEPES at pH 7 (each curve corresponds to the average of three consecutive measurements).

3. EuL1 assay

3.1 General procedure for transport measurements with EuL1 assay

Quartz cuvettes containing 3.00 mL of LUVs suspension with the probe $[Eu.L^1]^+$ encapsulated, were placed in the sample compartment of an Agilent Cary Eclipse fluorescence spectrometer equipped with a xenon flash lamp, a magnetic stirrer, and a temperature controller. Excitation and emission filters (ex.: 250 – 395 nm, em.: 430 – 1100 nm) were employed. The samples were excited at 332 nm (20 nm slits) and the emission intensity at 615 nm (5 nm slits) was monitored over time. A time-gate was applied (0.1 ms delay, acquisition time 2 ms, total decay time 3 ms). Three transport experiments were run in parallel.

Transporters **1** and **2**, monensin (**M**), valinomycin (**V**), and carbonyl cyanide 3-chlorophenylhydrazone (**CCCP**) were added as 5 µL of their stock solutions in methanol to reach the desired transporter to lipids molar ratio. Liposome suspensions were stirred for 3 minutes to stabilize the sample temperature to 25 ˚C. While the sample was stirring, a pulse of NaF (50 µL, 0.18 M NaF in 225 mM NaCl and 0.5 mM HEPES) was added to the vesicle sample to reach a total F^- concentration of 3 mM. 0.18 M KF in 225 mM KCl and 0.5 mM HEPES solution was used for the studies in KCl and K^+Gluconate$^-$. After 600 seconds, a detergent (Triton X-100, 50 µL of 5% w/w in H_2O) was added to lyse the liposomes.
The normalised transport curves were obtained using the equation:

$$Normalised\ Emission\ Intensity = \frac{E_t - E_0}{E_f - E_0}$$

Where E_t is the emission intensity at time t, E_0 is the emission intensity at $t = 0$, and E_f is the final emission intensity after the addition of the detergent.

3.2 Optimization of the EuL1 assay

HEPES concentration

LUVs containing 50 µM of the probe $[Eu.L^1]^+$ and NaCl 225 mM were buffered at pH 7 using different concentrations of HEPES to investigate the effect of the buffer concentration on the HF diffusion into the liposomes in absence of a transporter.

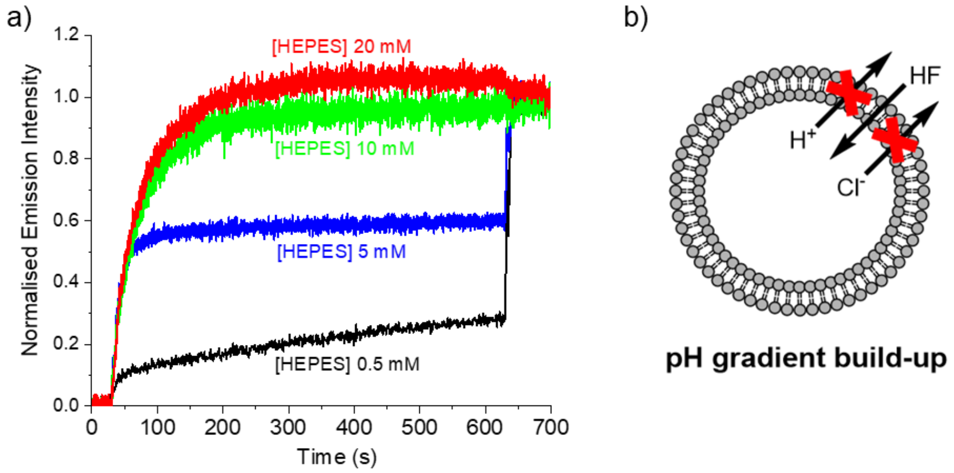

Figure S3. a) Normalised transport curves upon addition of NaF (3 mM) in absence of transporters at different HEPES concentration. **b)** Schematic representation of the mechanism causing the build-up of a pH gradient across the lipidic membrane of liposomes. These experiments were performed using a Fluoromax-4 spectrometer, see Section 4.3 for more details.

The HF diffusion is related to the concentration of buffer used. When 10 mM HEPES (or higher) is used, complete equilibration of fluoride concentrations is reached. The use of 5 mM HEPES decreases the HF influx, but a significant initial increase of emission is still observed before reaching a plateau, due to the build-up of a pH gradient that stops the further HF diffusion into the liposomes. Further lowering the concentration of HEPES to 0.5 mM minimises the HF diffusion across the membrane in absence of transporters, while still allowing to control the pH of the buffer solution at the start of the experiments. For these reasons, a HEPES concentration of 0.5 mM was used for all experiments with the EuL1 assay.

Pulse concentration

The response of the probe $[Eu.L^1]^+$ upon increasing concentrations of fluoride is based on the strong coordination of this anion to the Eu^{3+} cation. To avoid saturation of the probe (and thus a reduced sensitivity to changes in the fluoride concentration), but still ensuring a clear response of the $[Eu.L^1]^+$ probe, an experiment was performed to optimise the concentration of the NaF pulse added to the liposome suspension.

Liposomes were prepared as described in Section 2a and transporter **2** was post-inserted in the lipidic membrane of the liposomes via the addition of 5 µL of a 24 µM stock solution of **2** in methanol (to reach 0.01 mol%). Consecutive NaF pulses were added at intervals of circa 90 seconds to ensure completion of transport. Addition of 3 mM NaF results in a clear response while avoiding saturation, which is reached after addition of ~20 mM NaF. Given the results obtained, 3 mM F^- was used as pulse for all the experiments employing the EuL1 assay.

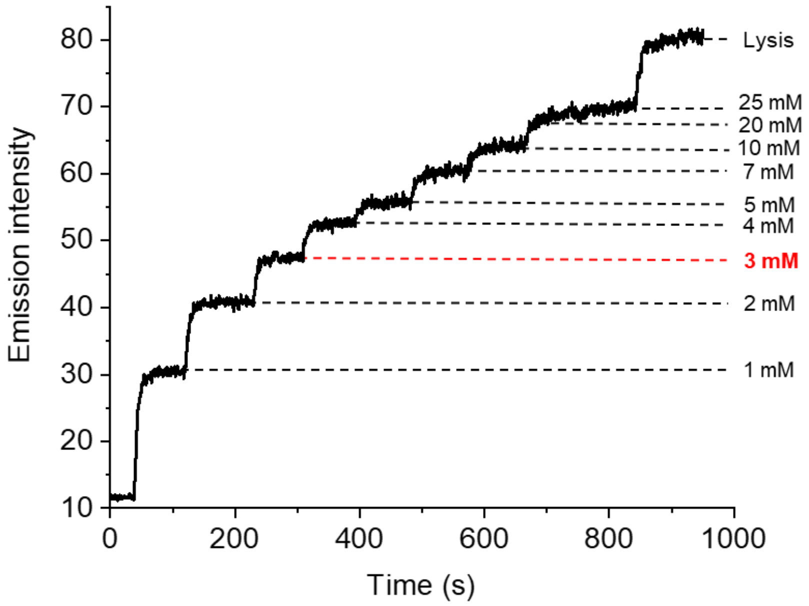

Figure S4. Transport of F^- mediated by transporter **2** (post-inserted at 0.01 mol%) across the lipidic membrane of LUVs monitored by the EuL1 assay in 225 mM NaCl buffered at pH 7 using 0.5 mM HEPES, upon multiple additions of NaF.

Probe response

In order to have an idea of the rate of equilibration of the emission intensity of the $[Eu.L^1]^+$ probe (without encapsulation in liposomes) upon addition of fluoride, a KF pulse (3 mM) was added to a solution containing the probe (50 nM in KCl 225 mM, HEPES 0.5 mM, pH 7). As shown in Figure S5, the increase of emission was imminent, and equilibration was reached within 5 seconds. This timescale of a few seconds is more likely to reflect the time it takes to fully mix the solutions in the cuvette than the time it takes for the probe to bind the fluoride anion. The observed time for equilibration in this experiment is shorter than the equilibration time in any of the transport experiments, where a datapoint is recorded every 8 seconds.

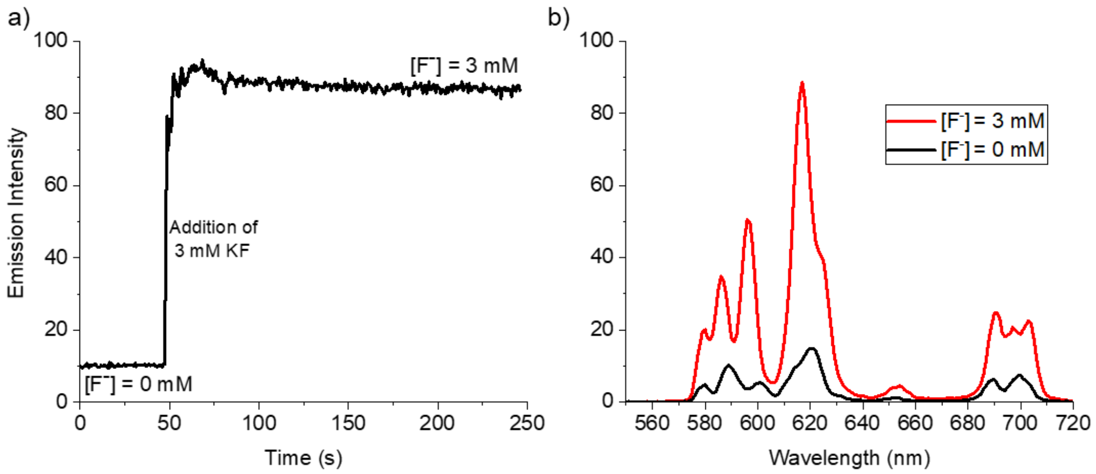

Figure S5. a) Control experiment to show the response of the $[Eu.L^1]^+$ probe to fluoride. KF (3 mM) was added at 47 seconds to an aqueous solution of the probe (50 nM in KCl 225 mM, HEPES 0.5 mM, pH 7). The curve was obtained monitoring the emission intensity at 615 nm upon excitation at 332 nm with application of time-gate. **b)** Emission spectra of $[Eu.L_1{}^+]$ before (black line) and after (red line) addition of 3 mM KF.

3.3 Quantification of rates of transport and half-lives.

Origin 2019b was used to fit the transport curves (average of three experiments, normalised), considering the data measured between 30 seconds (addition of NaF pulse) and 530 seconds (after which the data were removed).
To determine the half-life ($t_{½}$), the normalised transport curves were fitted to a single exponential function (YldFert1):

$$y = a + be^{-kx}$$

The half-life $t_{½}$ was then calculated from fit parameter k using the equation:

$$t_{½} = \frac{\ln(2)}{k}$$

To obtain the initial rates of transport (I), the normalised curves were fitted to a double exponential function:

$$F = y - ae^{-bt} - ce^{dt}$$

Initial values for the fit parameters were used as follows: y = 1, a = 0.8, b = 0.05, c = 0.2, d = 0.01. The initial rate was calculated from fitted parameters a, b, c, and d using the equation:

$$I = ab + cd$$

Table S2. Rate constants, half-lives and initial rates of transporters **1-3** in the EuL1 assay to monitor the F^-/Cl^- antiport in NaCl.

Transporter	Concentration (mol%)	Rate constant (s^{-1})	Half-life (s)	Initial rate (s^{-1})
**1**	0.1	0.016	43	0.063
**2**	0.001	0.029	24	0.104
**3**	0.1	0.005	150	0.011

3.4 EC_{50} calculation in the EuL1 assay to monitor F^-/Cl^- antiport

The transport curves for transporters **1** and **2** were measured at different concentrations. The EC_{50} values were determined via a Hill plot analysis of the emission intensity at 630 s (after 600 s of transport) plotted against the compound concentration. An EC_{50} of 0.009 mol% was found for **1** and 0.000014 mol% for **2**.

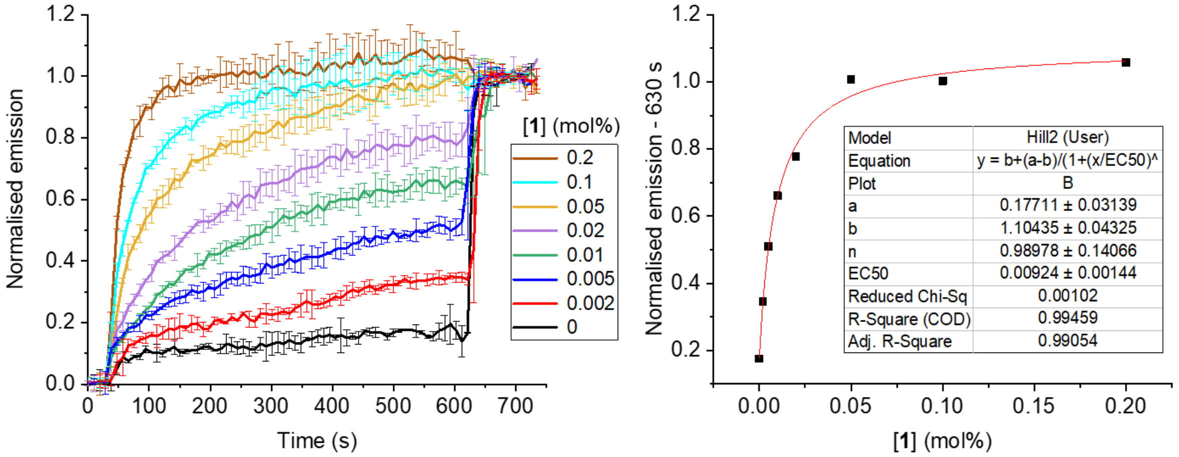

Figure S6. Hill plot analysis of the transport activity of compound **1** in the EuL1 assay to monitor F^-/Cl^- antiport upon addition of NaF (3 mM, after 30 s) and prior to lysis (after 630 s). The transporter was added as solution in methanol 3 minutes before the start of the measurement and concentrations are shown as transporter to lipids molar percentage. Error bars represent standard deviations from three experiments.

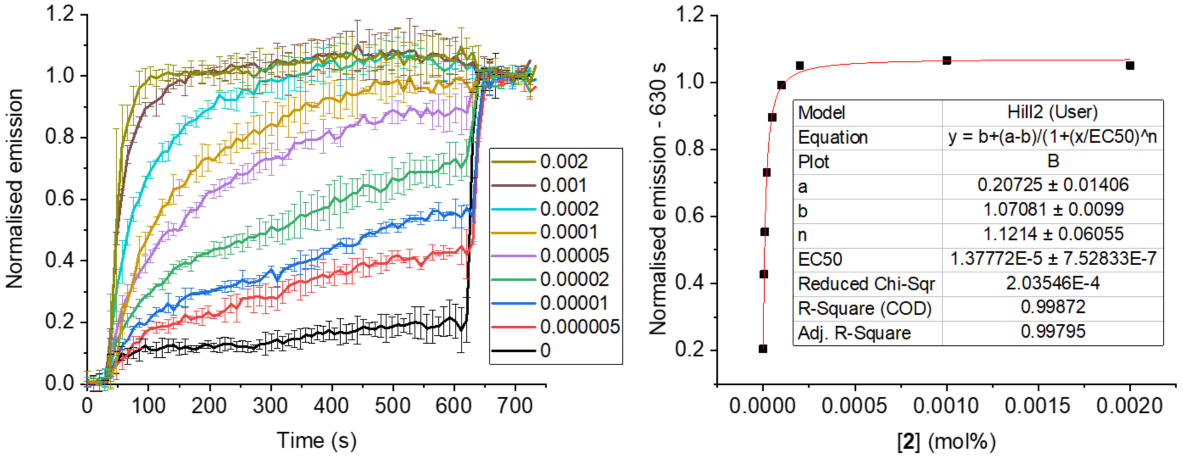

Figure S7. Hill plot analysis of the transport activity of compound **2** in the EuL1 assay to monitor F^-/Cl^- antiport upon addition of NaF (3 mM, after 30 s) and prior to lysis (after 630 s). The transporter was added as a solution in methanol 3 minutes before the start of the measurement and concentrations are shown as transporter to lipids molar percentage. Error bars represent standard deviations from three experiments.

3.5 EuL1 assay to monitor F^-/NO_3^- antiport

In order to study the ability of the transporters to work as F^-/NO_3^- antiporters, the LUVs suspension was prepared as in Section 2c ($NaNO_3$ is used instead of NaCl for preparation of the buffer) and the transport measurements were performed as described in Section 3.1.

The results in Figure S8 show activity for all the anion transporters. Although the conditions used encourage F^-/NO_3^- antiport, the observed response can be due to NO_3^-/OH^- antiport in combination with HF diffusion (as discussed in the main manuscript for the study of F^-/Cl^- antiport). This is demonstrated by the activity observed for monensin, which can be attributed to H^+/Na^+ antiport combined with HF diffusion.

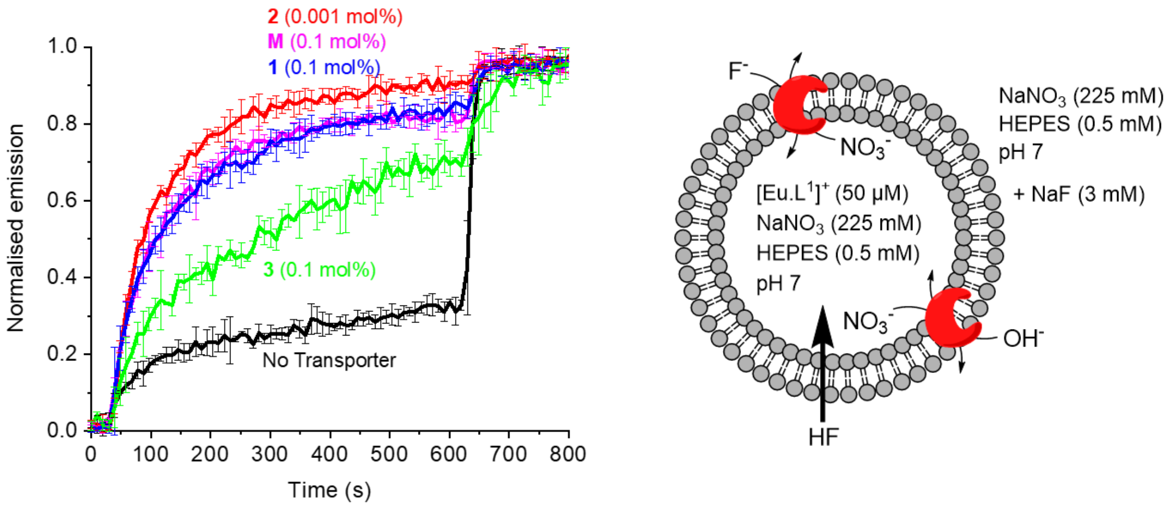

Figure S8. Transport curves obtained in the EuL1 assay to monitor F^-/NO_3^- antiport upon addition of NaF (3 mM, after 30 s) and prior to lysis (after 630 s). Transporters **1** and **2** and monensin (**M**) were added as solution in methanol 3 minutes before the start of the measurement. Transporter **3** was pre-incorporated in the membrane during the preparation of the liposomes The transporters concentrations are shown as transporter to lipids molar percentage. Error bars represent standard deviations from three experiments.

3.6 Study of transmembrane transport of fluoride in potassium chloride

In order to assess the effect of different cations on the fluoride transport activity of compounds **1**-**3**, the LUVs suspension was prepared as in Section 2b (KCl was used instead of NaCl for the preparation of the buffer) and the transport measurements were performed as described in Section 3.1.
The results in Figure S9 show that the HF diffusion response in absence of a transport is higher in KCl compared to NaCl, but the trends observed for transporters **1**-**3** and the corresponding half-lives are similar (66 s^{-1} for **1**, 13 s^{-1} for **2** and 103 s^{-1} for **3** in KCl) to the values obtained in NaCl (Section 3.3, Table S2). Thus, we concluded that the effect of the counter cation can be considered negligible.

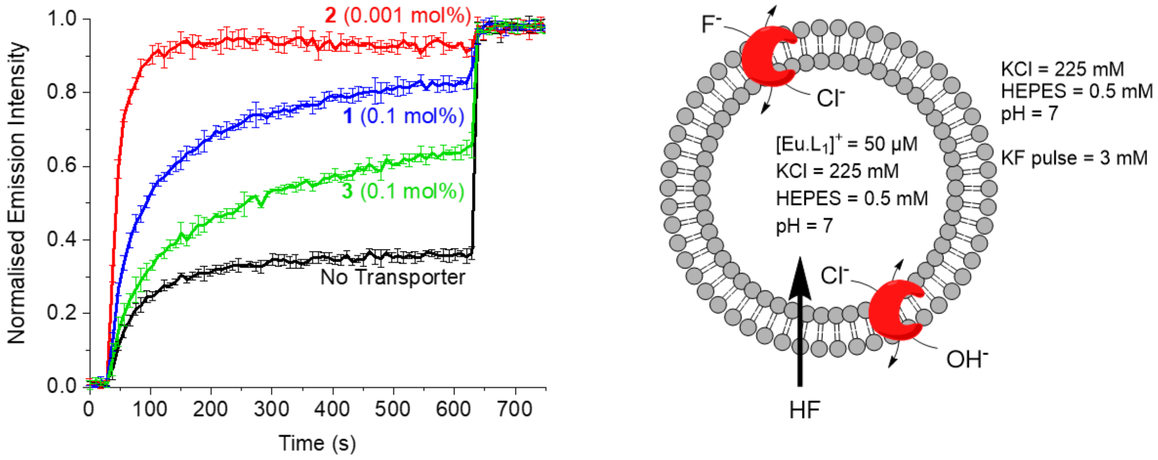

Figure S9. Transport curves obtained in the EuL1 assay to monitor F^-/Cl^- antiport and assess the possibility of F^-/K^+ symport upon addition of KF (3 mM, after 30 s) and prior to lysis (after 630 s). Transporters **1** and **2** were added as solution in methanol 3 minutes before the start of the measurement. Transporter **3** was pre-incorporated in the membrane during the preparation of the liposomes The transporters concentrations are shown as transporter to lipids molar percentage. Error bars represent standard deviations from three experiments.

3.7 EuL1 assay to monitor F^- uniport

In these F^- uniport experiments, the readily transportable anions Cl^- (or NO_3^-) are replaced by gluconate, whose high polarity impedes its transport. Furthermore, Na^+ is replaced by K^+, which can be efficiently transported across the membrane by the cationophore valinomycin (see Section 2d). The data are shown in Figure S10.

Transporter **3** (0.1 mol%) was active when tested in combination with valinomycin (0.02 mol%) and mild activity was recorded for **1** (0.1 mol%), while **2** was not able to perform F^- uniport at the concentration (0.001 mol%) that gave clear transport in NaCl and $NaNO_3$ solutions.

Although the conditions studied aimed at observing F^- uniport, a potential mechanism that could cause fluoride intake into the liposomes is OH^- influx (or H^+ efflux) in combination with HF diffusion. This was proven using protonophore carbonyl cyanide 3-chlorophenylhydrazone (**CCCP**, 0.1 mol%),[8] which in combination with valinomycin showed good transport activity. For this reason, transporters **1**, **2** and **3** were tested in a modified HPTS assay for monitoring OH^- uniport (refer to Section 5.6), but none of the compounds showed any significant activity. This suggests that the activity observed for **3** and **1** in Figure S10b is caused by actual F^- uniport.

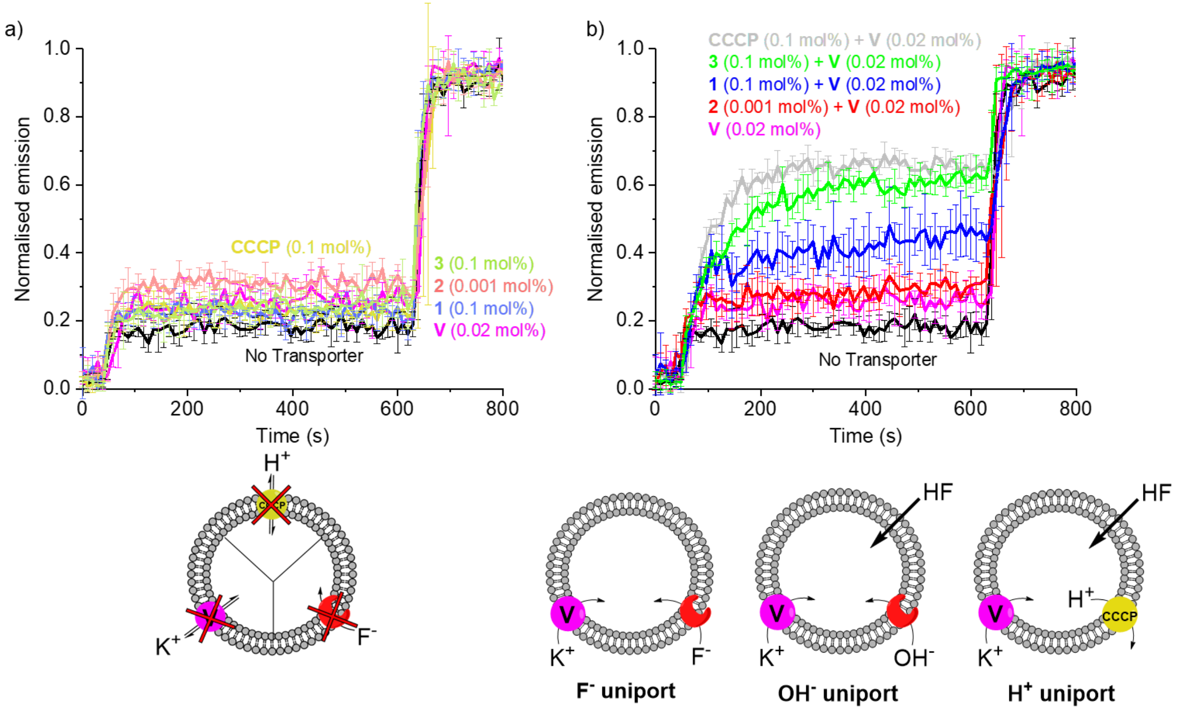

Figure S10. Transport curves obtained in the EuL1 assay to monitor F^- uniport upon addition of KF (3 mM, after 30 s) and prior to lysis (after 630 s). Transporters **1** and **2**, valinomycin (**V**) and **CCCP** were added as solution in methanol 3 minutes before the start of the measurement. Transporter **3** was pre-incorporated in the membrane during the preparation of the liposomes. The transporters are tested without **(a)** and with **(b)** valinomycin. The transporters concentrations are shown as transporter to lipids molar percentage. Error bars represent standard deviations from three experiments.

4. Lifetime measurements and application of time-resolved measurements

4.1 Lifetime of $[Eu.L^1]^+$ encapsulated in LUVs

A quartz cuvette containing 3.00 mL of LUVs suspension with probe $[Eu.L^1]^+$ encapsulated (prepared as in Section 2a) was placed in the sample compartment of a Agilent Cary Eclipse fluorescence spectrometer equipped with a xenon flash lamp, a magnetic stirrer and a temperature controller. Excitation and emission filters (ex: 250-395 nm, em: 430-1100 nm) were employed. Transporter **2** was added to the liposomes (5 µL of 0.024 mM to reach a concentration of 0.01 mol% transporter/lipid molar ratio) to ensure fast equilibration of fluoride across the liposomal membrane.
The suspension was stirred for 3 minutes to stabilize the sample temperature to 25 ˚C. Lifetime measurements were recorded in the presence of 0, 3, 10, and 25 mM fluoride. After each NaF addition, the emission intensity at 615 nm was monitored for a few minutes until constant emission intensity was reached, after which the lifetime measurement was performed using the following parameters:

- Ex : 332 nm (slits : 20 nm)
- Em : 615 nm (slits : 20 nm)
- Delay time: 0.1 ms
- Acquisition time: 0.05 ms
- Total decay: 10 ms

Origin was used to plot the obtained decay curves, which were fitted with the equation:

$$I_N = A_0 + A_1 e^{-kt}$$

Where I_N is the normalised emission intensity at time t following excitation, A_0 is intensity when decay has ceased, A_1 is the pre-exponential factor, and k is the rate constant for the depopulation of the excited state.

The emission lifetime (τ) of the probe $[Eu.L^1]^+$ was calculated as:

$$\tau = \frac{1}{k}$$

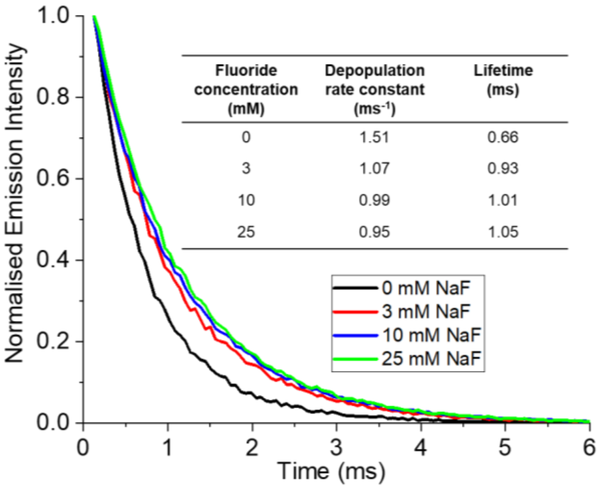

Figure S11. Emission decay curves of $[Eu.L^1]^+$ (50 µM) encapsulated in LUVs in presence of different amounts of NaF. The lifetime of the probe increases in presence of fluoride.

4.2 Lifetime of compound **1** incorporated in liposomes

The excited-state lifetime of transporter **1** was studied using a LP920-K spectrometer from Edinburgh Instruments. The excitation source was a tunable Nd:YAG Laser NT342 Series from EKSPLA. The third harmonic (355 nm) was attenuated to 10 mJ/pulse and was used to excite the sample. Luminescence lifetime was measured using a PMT-900 detected with a minimum detector response width of 5 ns. The instrument response function (IRF) was used to correct the measured excited-state lifetime. An average of 30 laser pulses were used to record excited-state lifetimes. Origin was used to plot and fit the obtained data and to calculate the lifetime as described in Section 4.1.

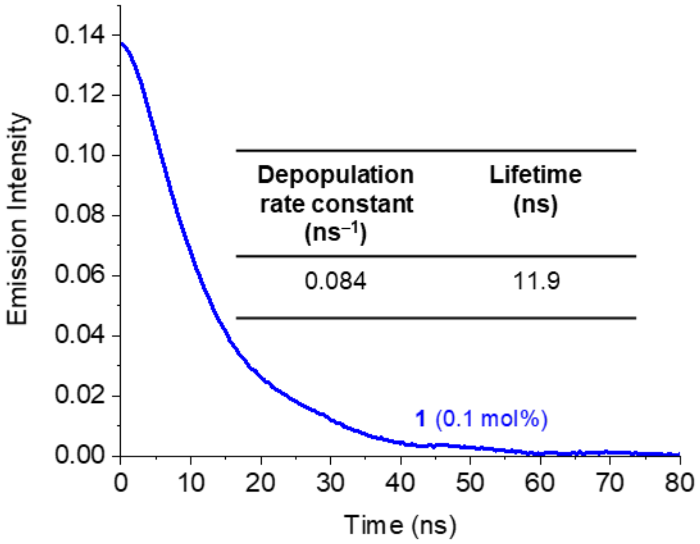

Figure S12. Emission decay of transporter **1** (0.1 mol%) incorporated in the lipid bilayer of LUVs prepared as in Section 2a.

4.3 EuL1 assay to study fluoride transport without time-gating

While the EuL1 assay allows the use of time-resolved fluorescence spectroscopy to study transport by fluorescent compounds, a regular fluorescence spectrometer with a continuous light source can also be used to study fluoride transport by this EuL1 assay, as demonstrated below.

A quartz cuvette containing 3.00 mL of LUVs suspension with probe $[Eu.L^1]^+$ encapsulated prepared as in Section 2a, was placed in the sample compartment of a Fluoromax-4 spectrometer, equipped with a 245-530 nm cut-off filter between the sample and the detector. Emission spectra were recorded from 550-720 nm (3 nm slits, 1 nm interval, 0.3 s integration time) with excitation at 330 nm (10 nm slits). During transport measurements the emission intensity at 615 nm (3 nm slits) was monitored over time (15 minutes, 0.2 s interval) with excitation at 330 nm (10 nm slits). 5 µL of a stock solution (2.4 µM) of transporter **2** in methanol were added to reach the desired transporter to lipids molar percentage (0.001 mol%).
Liposome suspensions were stirred for 3 minutes to stabilize the sample temperature to 25 ˚C. While the sample was stirring at 25 ˚C, a pulse of NaF (50 µL, 0.18 M) was added to reach a total F^- concentration of 3 mM. After 10 minutes, a detergent (Triton X-100, 50 µL of 5% w/w in H2O) was added to lyse the liposomes.

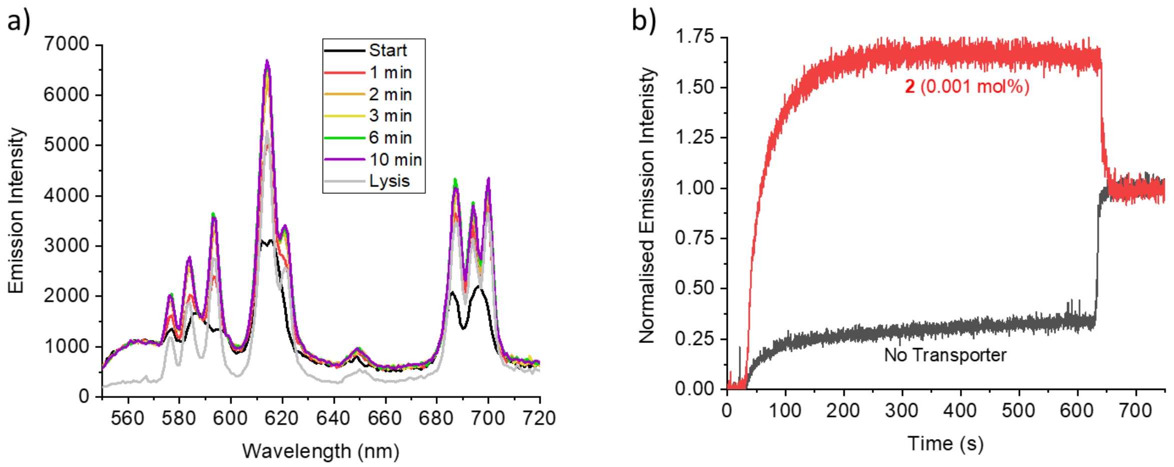

Figure S13. a) Emission spectra of $[Eu.L^1]^+$ recorded over time during transport by **2** (at 0.001 mol%) without the application of time-gate. **b)** Transport curves obtained in the EuL1 assay monitoring overtime the emission intensity at 615 nm obtained via excitation of LUVs (as in a) at 332 nm without application of time-gate. Transporter **2** was added as solution in methanol 3 minutes before the start of the measurement. The transporter concentration is shown as transporter to lipids molar percentage.

5. HPTS assay

5.1 HPTS experiment with NaF pulse to prove pH build-up

In order to understand the effect of the addition of NaF on the internal pH of the vesicles during a typical experiment using the EuL1 assay to monitor the transport of fluoride, LUVs were prepared as in Section 2a, but HPTS was encapsulated instead of $[Eu.L^1]^+$. Thus, the final system was composed of liposomes containing 1 mM HPTS and suspended in 225 mM NaCl, buffered at pH 7 using 0.5 mM HEPES and subjected to a 3 mM NaF pulse (Figure S14). In absence of transporters, a fast decrease of pH was observed (from 7.20 to 6.75) upon addition of NaF. This acidification is caused by the diffusion of HF into the vesicles, leading to the build-up of a pH gradient. This gradient disappears upon lysis.

In presence of transporter **2** (0.001 mol%) after a slight acidification caused by initial HF diffusion into the liposomes, rapid equilibration of pH was observed, caused by the efficient Cl^-/OH^- antiport activity of the transporter (see Sections 5.3 and 5.4).

Vesicles with transporters **1** and **3** (0.1 mol%) also show initial acidification, followed by a pH equilibration slower than by **2**. In the case of **3**, which cannot work as Cl^-/OH^- antiporter (see Figure S16), the equilibration of pH is most likely caused by F–/Cl– antiport. In this case, after initial pH build-up caused by fast HF diffusion into the liposomes, the F^-/Cl^- antiport by **3** can induce a reverse HF diffusion (from inside to outside) to dissipate the pH gradient.

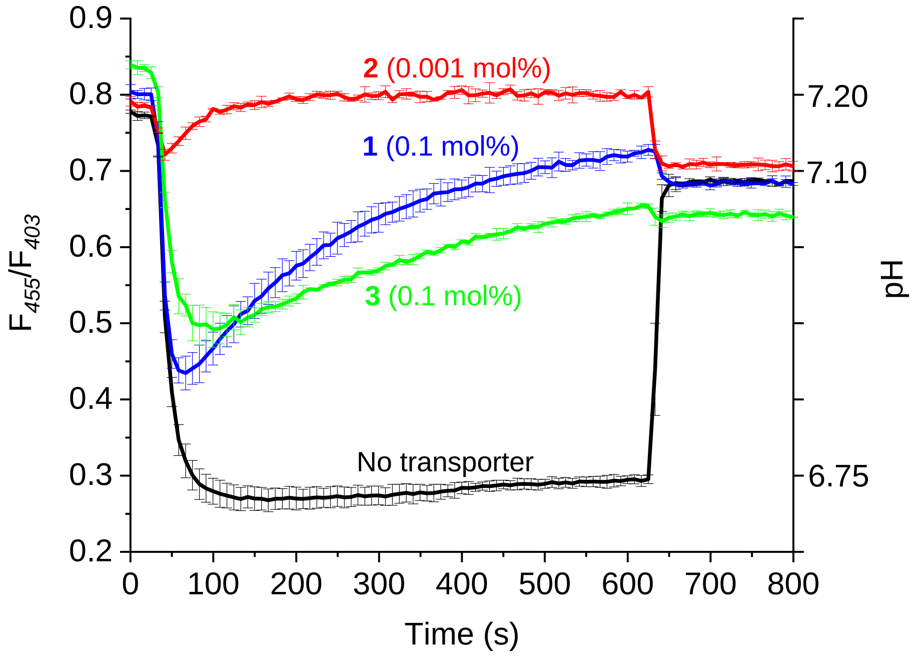

Figure S14. Control experiment to monitor the effect of a NaF (3 mM) external pulse to the internal pH of LUVs containing HPTS (1 mM) and NaCl (225 mM) and HEPES (0.5 mM) at pH 7 suspended in NaCl (225 mM) and HEPES (0.5 mM). Transporters **1** and **2** were added as solution in methanol 3 minutes before the start of the measurement. Transporter **3** was pre-incorporated in the membrane during the preparation of the liposomes. The LUVs were lysed 600 seconds after the addition of NaF. The transporters concentrations are shown as transporter to lipids molar percentage. Error bars represent standard deviations from three experiments.

To calibrate the response of intravesicular HPTS to pH, monensin (0.1 mol%) was added to 3 mL of a solution of vesicles containing 1 mM HPTS, 225 mM NaCl, 0.5 mM HEPES and buffered at pH 7. The pH of the sample was modified by adding HCl or NaOH and monitored using a pH-meter. The sample was stabilized (stirring, 25°C). Then the excitation spectrum was recorded using an Agilent Cary Eclipse fluorescence spectrometer (em: 511 nm, slits 5 nm, 5 nm).

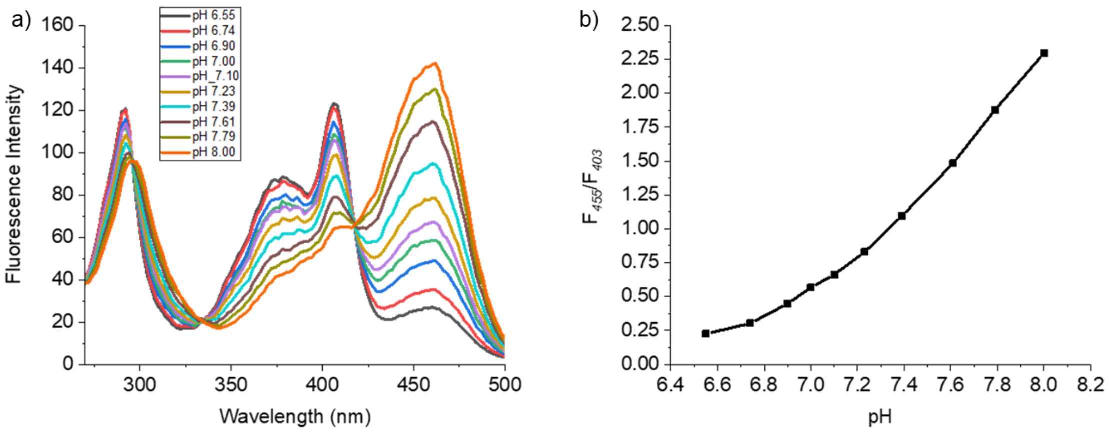

Figure S15. a) Excitation spectra of HPTS encapsulated in LUVs at different pH (emission at 511 nm). **b)** Graph of the fluorescence ratio ex(455nm)/ex(403nm) against external pH (measured with a pH-meter).

5.2 General procedure for transport measurements with HPTS

Liposomes were prepared as described in Sections 2e and 2f. Quartz cuvettes containing 3.00 mL of the LUVs suspension with dye HPTS (1 mM ex: 403 nm and 455 nm, em: 511 nm) encapsulated were placed in the sample compartment of an Agilent Cary Eclipse fluorescence spectrometer equipped with a xenon flash lamp, a magnetic stirrer, and a temperature controller. Three transport experiments were run in parallel. If transporters **1** and **2**, monensin or carbonyl cyanide 3-chlorophenylhydrazone (CCCP, serving as protonophore) were used, these were added in 5 µL of their stock solutions in methanol to reach the desired transporter to lipids ratio and the suspension was stirred for 3 minutes to stabilize the sample temperature to 25 ˚C. For the study of OH^-/Cl^- antiport and Cl^- uniport, while the sample was stirring at 25 ˚C, a base pulse of NMDG (5 mM) was added to the sample to generate a pH gradient between the inside (pH 6.8) and the outside (pH 7.8) of the vesicles as prepared as in Section 2e. For the study of OH^- uniport, the base pulse consisted of the addition of tetrabutylammonium hydroxide (TBAOH, 5 mM) to the vesicles prepared as in Section 2f.
The rate of the pH gradient dissipation was monitored by recording the ratio between the fluorescence emissions of the protonated (excitation at 403 nm) and deprotonated (excitation at 455 nm) forms of HPTS (F_{455}/F_{403}). After 200 seconds, a detergent (Triton X-100, 50 µL of 5% w/w in H_2O) was added to lyse the liposomes and dissipate the pH gradient. The ratio of the fluorescence intensities was normalised using the following equation:

$$Normalised\ curves = \frac{R_t - R_0}{R_f - R_0}$$

Where R_t is the fluorescence ratio at time t, R_0 is the fluorescence ratio at t = 0, and R_f is the final fluorescence ratio after the addition of the detergent.

5.3 HPTS assay to monitor OH^-/Cl^- antiport and Cl^- uniport

To test the ability of the transporters to work as OH^-/Cl^- antiporters, the HPTS assay was employed. LUV suspensions were prepared as described in Section 2e and results are shown in Figure S16.

In the absence of CCCP, the increase of F_{455}/F_{403} is caused by the ability of the transporter to perform OH^-/Cl^- antiport. While transporter **1** showed mild transport activity when tested at 0.1 mol%, **2** was able to work as OH^-/Cl^- antiporter at very low concentration (0.0001 mol%, see also Figure S17). On the other hand, **3** did not show activity when tested at 0.1 mol%, as a result of its inability to work as OH^-/Cl^- antiporter.

In combination with protonophore CCCP (0.1 mol%), a slight increase of transport activity was recorded for **1**, as a result of a slight selectivity for Cl^- over OH^-. The combination of **2** and CCCP did not trigger any difference on the rate of transport, indicating that the transport of OH^- is not rate limiting. In contrast, when combined with CCCP, transporter **3** shows outstanding activity and it is clearly active as Cl^- uniporter.

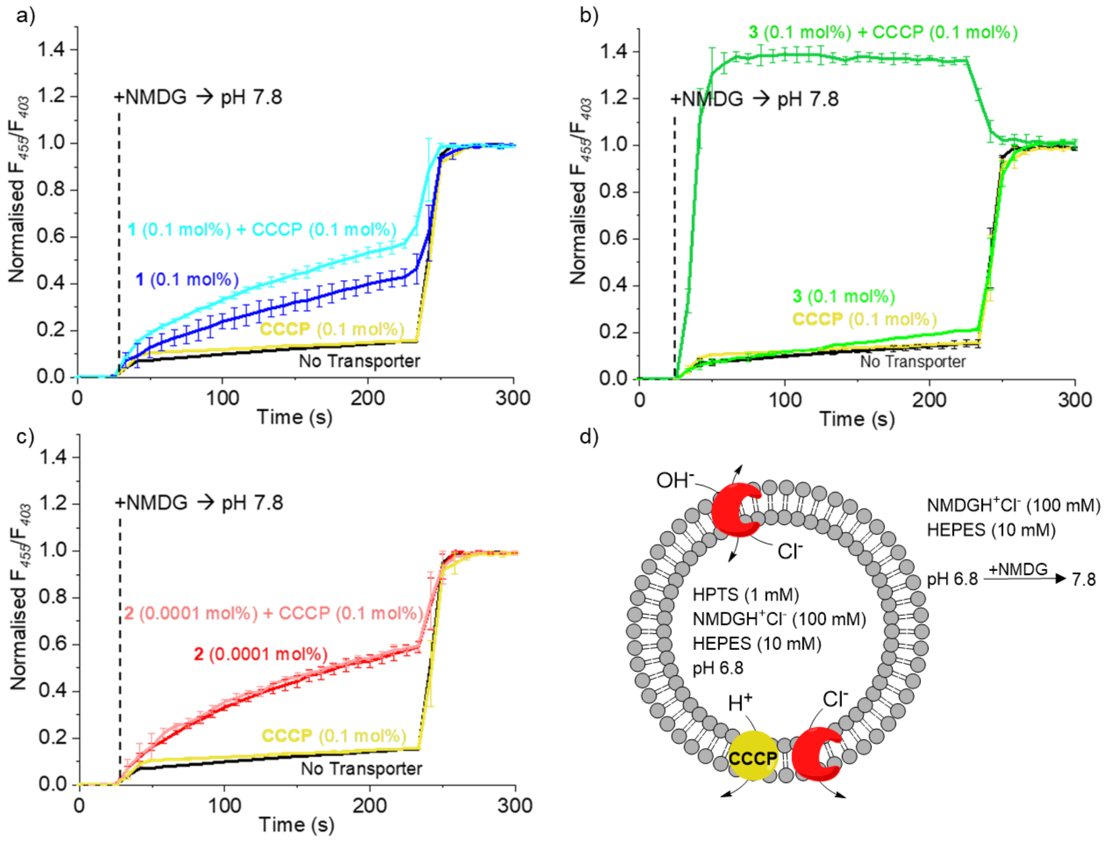

Figure S16. Dissipation of a pH gradient mediated by transporters **1** (a), **2** (b) and **3** (c) monitored via the HPTS assay to monitor OH^-/Cl^- antiport (in absence of CCCP) and Cl^- uniport (in presence of CCCP) upon addition of NMDG (5 mM, after 30 s) and prior to lysis (after 230 s). Liposomes prepared as in Section 2e. Transporters **1** and **2** and CCCP were added as solution in methanol 3 minutes before the start of the measurement. Transporter **3** was pre-incorporated in the membrane during the preparation of the liposomes. The transporters concentrations are shown as transporter to lipids molar percentage. Error bars represent standard deviations from three experiments.

5.4 EC_{50} calculation in the HPTS assay to monitor the OH^-/Cl^- antiport

The EC_{50} value for transporter **2** as OH^-/Cl^- antiporter was determined via a Hill plot analysis, performed with the transporter post-inserted at different concentrations and the normalised fluorescence ratio at 230 s (after 200 s of transport) plotted against the compound concentration (Figure S17). An EC_{50} value of 0.00015 mol% was obtained.

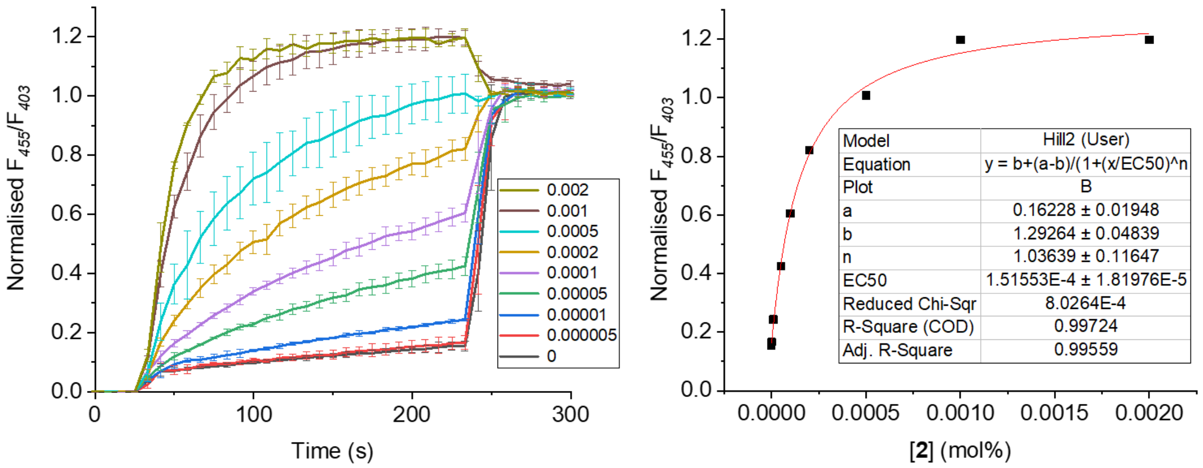

Figure S17. Hill plot analysis of the transport activity of compound **2** in the HPTS assay to monitor OH^-/Cl^- antiport upon addition of NMDG (5 mM, after 30 s) and prior to lysis (after 230 s). The transporter was added as a solution in methanol 3 minutes before the start of the measurement. The transporter concentrations are shown as transporter to lipids molar percentage. Error bars represent standard deviations from three experiments.

5.5 Comparison of the EuL1 and HPTS assays

The similar conditions used in the EuL1 assay for the study of F^-/Cl^- antiport and in the HPTS assay for the study of OH^-/Cl^- antiport enabled the direct comparison of results from the two assays via plotting the normalised transport curves against time (230 s) together. This comparison is presented in Figure 4 of the main text and Figure S18 shows the activity of **2** at 0.001 mol%, showing similar curves in both assays.
However, as the curves from both assays reach equilibration rather fast at this concentration of **2**, a lower concentration is used in Figure 4b, allowing to better assess the relative contributions of the different transport mechanisms to the response in the EuL1 assay.

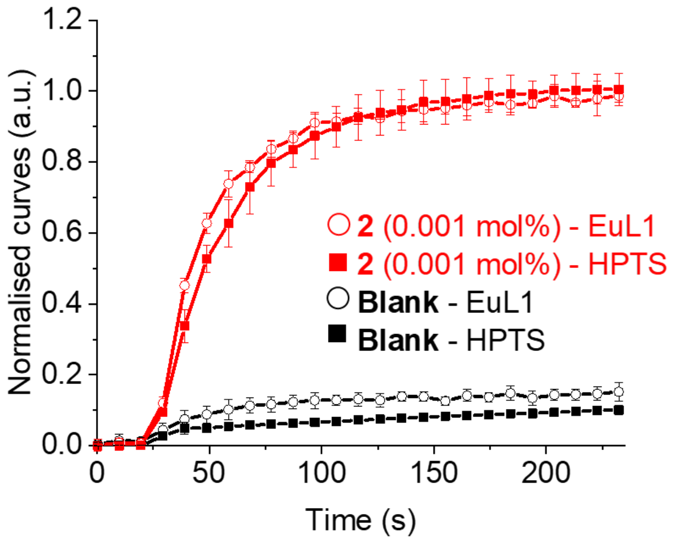

Figure S18. Direct comparison of transport curves obtained in the EuL1 (open symbols) and HPTS (filled symbol) assays for transporter **2** tested at 0.001 mol%.

5.6 HPTS assay to monitor OH^- uniport

In order to test the ability of the transporters to work as OH^- uniporters, the HPTS assay was employed. LUV suspensions were prepared as described in Section 2f.
This experiment was a crucial control experiment related to the ability of transporters **1** and **3** to work as F^- uniporters discussed in Section 3.7. While the experiments are performed in the same way as described for the study of OH^-/Cl^- antiport (Section 5.3), some modifications are applied to the system used. The $NMDGH^+Cl^-$ is replaced by sodium gluconate and NMDG is replaced by TBAOH. The free diffusion of the tetrabutylammonium cation (TBA^+) across the lipidic membrane balances the transport of OH^-, while gluconate anions are too polar to diffuse or to be transported through the membrane.[9] As a result, the only mechanism that can generate a response in these conditions is the uniport of OH^- (or H^+) mediated by the transporter.
Figure S19 shows no significant transport by any of the compounds. This lack of activity in OH^- uniport observed for **1** and **3**, proved that the response obtained in the EuL1 assay was caused by F^- uniport. On the other hand, compound **2** was unable to work as neither OH^- nor F^- uniporter at the concentration tested.

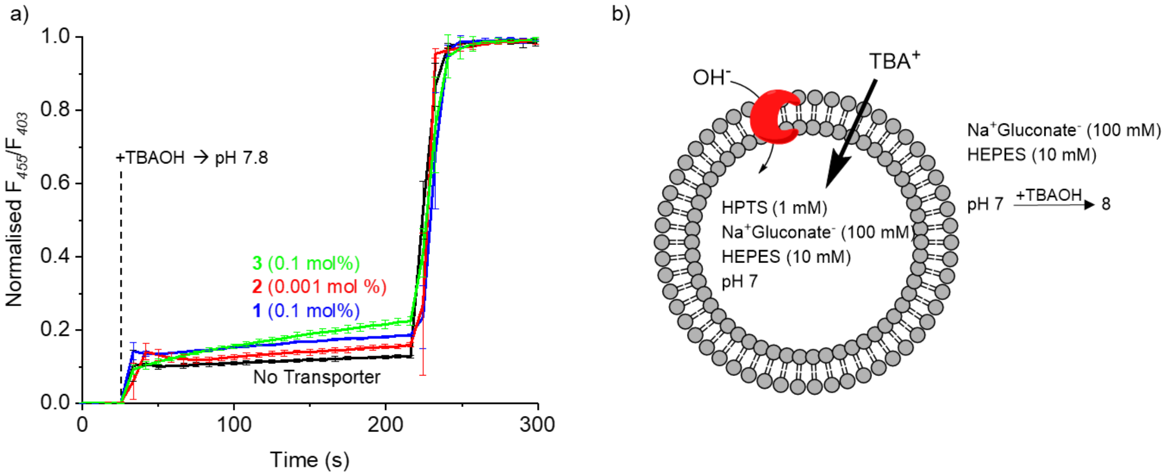

Figure S19. Dissipation of a pH gradient mediated by transporters **1**, **2** and **3** monitored via HPTS assay to monitor OH^- uniport upon addition of TBAOH (5 mM, after 30 s) and prior to lysis (after 230 s). Liposomes were prepared as in Section 2f. Transporters **1** and **2** were added as solution in methanol 3 minutes before the start of the measurement. Transporter **3** was pre-incorporated in the membrane during the preparation of the liposomes. The LUVs were lysed after 200 seconds from the addition of TBAOH. The transporter concentrations are shown as transporter to lipids molar percentage. Error bars represent standard deviations from three experiments.

6. Application of the time-resolved EuL1 assay for the study of bicarbonate transport

The EuL1 assay can also be used for the study of HCO_3^- transport, as described in reference 10. In this previous report, a regular fluorescence spectrometer with continuous light source was used. However, the time-resolved measurements as described for the study of F^- transport in the current work, can also be applied for HCO_3^- transport studies.

For instance, the concentration of prodigiosin in reference 10 was limited to 0.004 mol% (1:25000), because at higher concentrations the fluorescence of prodigiosin interfered with the emission of the $[Eu.L^1]^+$. Thus, time-resolved measurements were applied to monitor the transport of HCO_3^- by prodigiosin at higher concentration (0.04 mol%) and the results are given in Figure S20.

These results show that a 10-fold higher concentration of prodigiosin (0.04 mol% instead of 0.004 mol%) does not lead to any faster increase of the HCO_3^- concentration inside the liposomes. This indicates that CO_2 diffusion is rate-limiting in the transport process and that prodigiosin transports HCO_3^- via a combination of HCl transport and CO_2 diffusion rather than HCO_3^-/Cl^- antiport,[10,11] even at a concentration of 0.04 mol%.

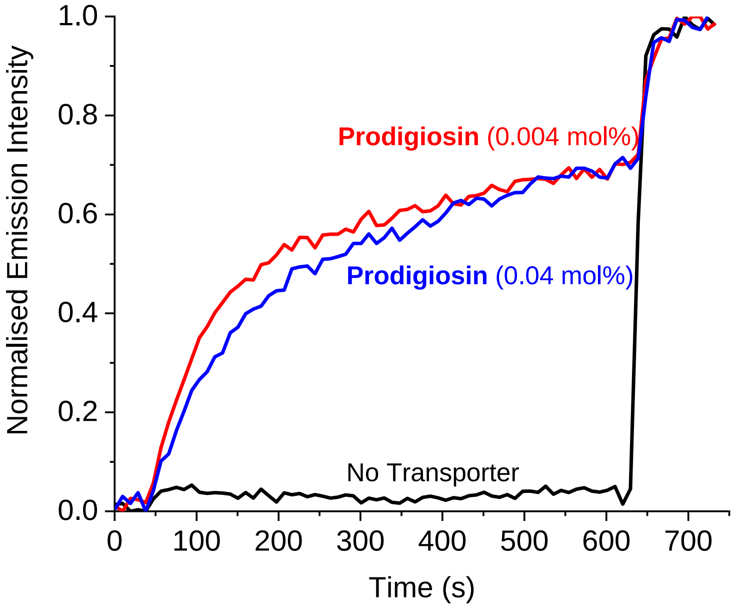

Figure S20. Transport curves obtained in the time-resolved EuL1 assay to monitor HCO_3^-/Cl^- antiport upon addition of $NaHCO_3$ (10 mM, after 30 s) and prior to lysis (after 630 s). Prodigiosin was added as a solution in methanol 3 minutes before the start of the measurement. The transporter concentrations are shown as transporter to lipids molar percentage.

References

[1] H. J. Clarke, E. N. W. Howe, X. Wu, F. Sommer, M. Yano, M. E. Light, S. Kubik and P. A. Gale, *J. Am. Chem. Soc.* 2016, 138, 16515–16522.

[2] G. Park, D. J. Brock, J.-P. Pellois and F. P. Gabbaï, *Chem* 2019, **5**, 2215–2227.

[3] G. Park and F. P. Gabbaï, *Angew. Chem. Int. Ed.* 2020, **59**, 5298–5302.

[4] V. M. Gonzalez, G. Park, M. Yang and F. P. Gabbaï, *Dalton Trans.* 2021, **50**, 17897–17900.

[5] I. S. Ke, M. Myahkostupov, F. N. Castellano and F. P. Gabbaï, *J. Am. Chem. Soc.*, 2012, 134, 15309–15311.

[6] H. Valkenier, O. Akrawi, P. Jurček, K. Sleziaková, T. Lízal, K. Bartik, V. Šindelář, *Chem* 2019, **5**, 429–444.

[7] S. J. Butler, Chem. Commun., 2015, **51**, 10879–10882.

[8] S.G. McLaughlin, J. P. Dilger, Physiol. Rev. 1980, **60**, 825-863

[9] X. Wu, L. W. Judd, E. N. W. Howe, A. M. Withecombe, V. Soto-Cerrato, H. Li, N. Busschaert, H. Valkenier, R. Pérez-Tomás, D. N. Sheppard, Y. B. Jiang, A. P. Davis and P. A. Gale, *Chem*, 2016, **1**, 127–146.

[10] L. Martínez-Crespo, S. H. Hewitt, N. A. De Simone, V. Šindelář, A. P. Davis, S. J. Butler and H. Valkenier, *Chem. - A Eur. J.*, 2021, 27, 7367–7375.

[11] K. Maslowska-Jarzyna, A. Cataldo, A. Marszalik, I. Ignatikova, S. J. Butler, R. Stachowiak, M. J. Chmielewski, H. Valkenier, *Org. Biomol. Chem.*, 2022, **20**, 7658-7663
